# Supplementary material for: Gut Microbiome Changes Occurring with Norovirus Infection and Recovery in Infants Enrolled in a Longitudinal Birth Cohort in Leon, Nicaragua
Source: Viruses. 2022 Jun 27;14(7):1395. doi: 10.3390/v14071395 (PMC9323674; doi:10.3390/v14071395)
Supplement: Supplementary file 1 [file viruses-14-01395-s001.zip › supplementary figures.pdf]

Supplemental material

Table S1. Extended characteristics of child and stool sample collection.

Table S2. Diversity and richness indices for gut microbiomes before, during and after norovirus AGE.

Table S3. Differential abundance of gut microbiome taxonomy at the genus level, comparing before and during disruptive norovirus AGE.

Table S4. Rank abundance of the top 20 species found in gut microbiomes.

Table S5. Differential abundance of gut microbiome taxonomy at the species level, comparing before and during disruptive norovirus AGE.

Table S6. Differential abundance of gut microbiome functional genes, comparing before and during disruptive norovirus AGE.

Table S7. Significantly different Tier 2 KEGG pathways found when comparing before and during microbiome disruption by norovirus AGE.

Table S8. Differential abundance of gut microbiome functional genes, comparing before and after disruptive norovirus AGE.

(**a**)
(b)

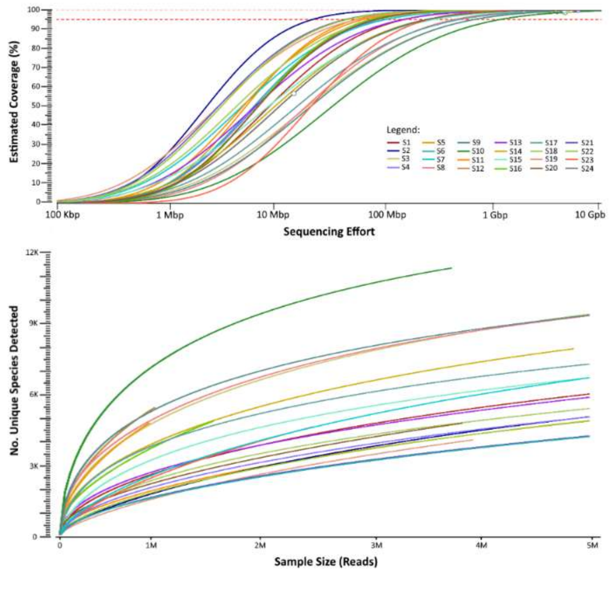

Figure S1. The breadth of coverage and species richness of genomes in the metagenome. Sufficient sequencing effort was obtained for statistical comparisons of gut microbiota, supported by a) the plateauing of curves representing the percent metagenome sequence coverage by sequencing effort for each sample, and b) the plateauing of rarefaction curves showing the number of unique species detected by number of sampled sequencing reads for each sample. Numbers following "S" indicate the sample identifier for metagenomic data. Colors indicate the samples obtained from each child (S1-S22) and positive (S23) and negative (S24) controls. Open circles on panel "a" show the sequencing effort for samples in this study, computed from kmer redundancy using reads (Nonpareil). Rarefaction curves are computed from delete-half jackknifing with 100 replicates.

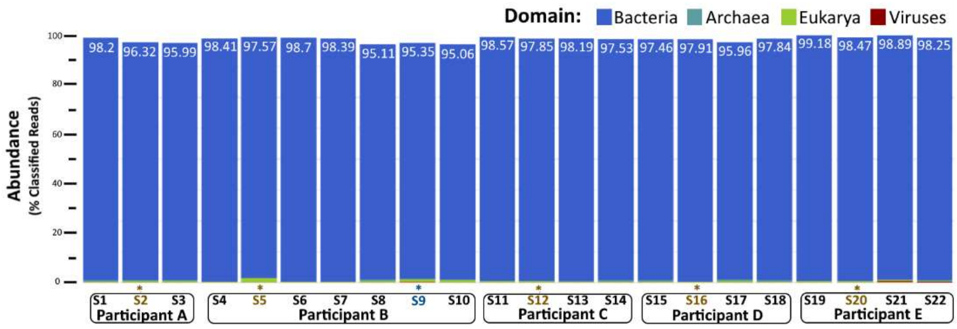

Figure S2. Gut microbiome changes in Domain-level composition before, during and after norovirus AGE. Nearly all classified reads were of the Bacterial Domain regardless of participant or stage of norovirus AGE (before, during and after). Brown colored text represents samples collected during norovirus AGE. For participant B, a second norovirus infection occurred indicated by blue text. Numbers following "S" indicate the sample identifier for metagenomic data.

(**a**)

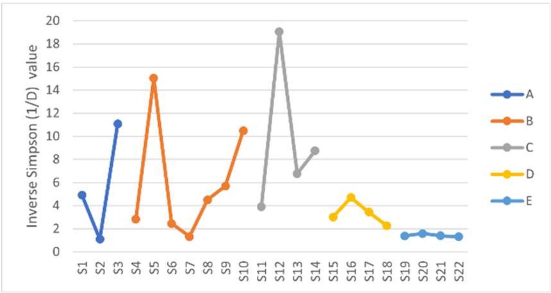

(**b**)

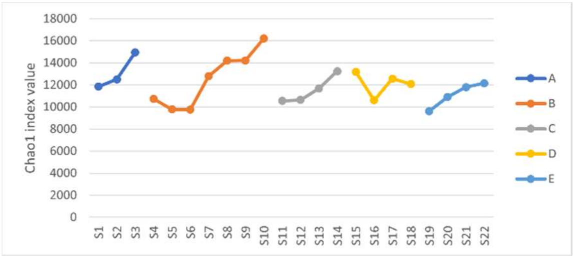

Figure S3. Gut microbiome changes in diversity and richness before, during, and after norovirus AGE, measured by a) Inverse Simpson (1/D) and b) Chao1. Numbers on the x-axis indicate the sample identifier for metagenomic data. Colored lines indicate the study participants.

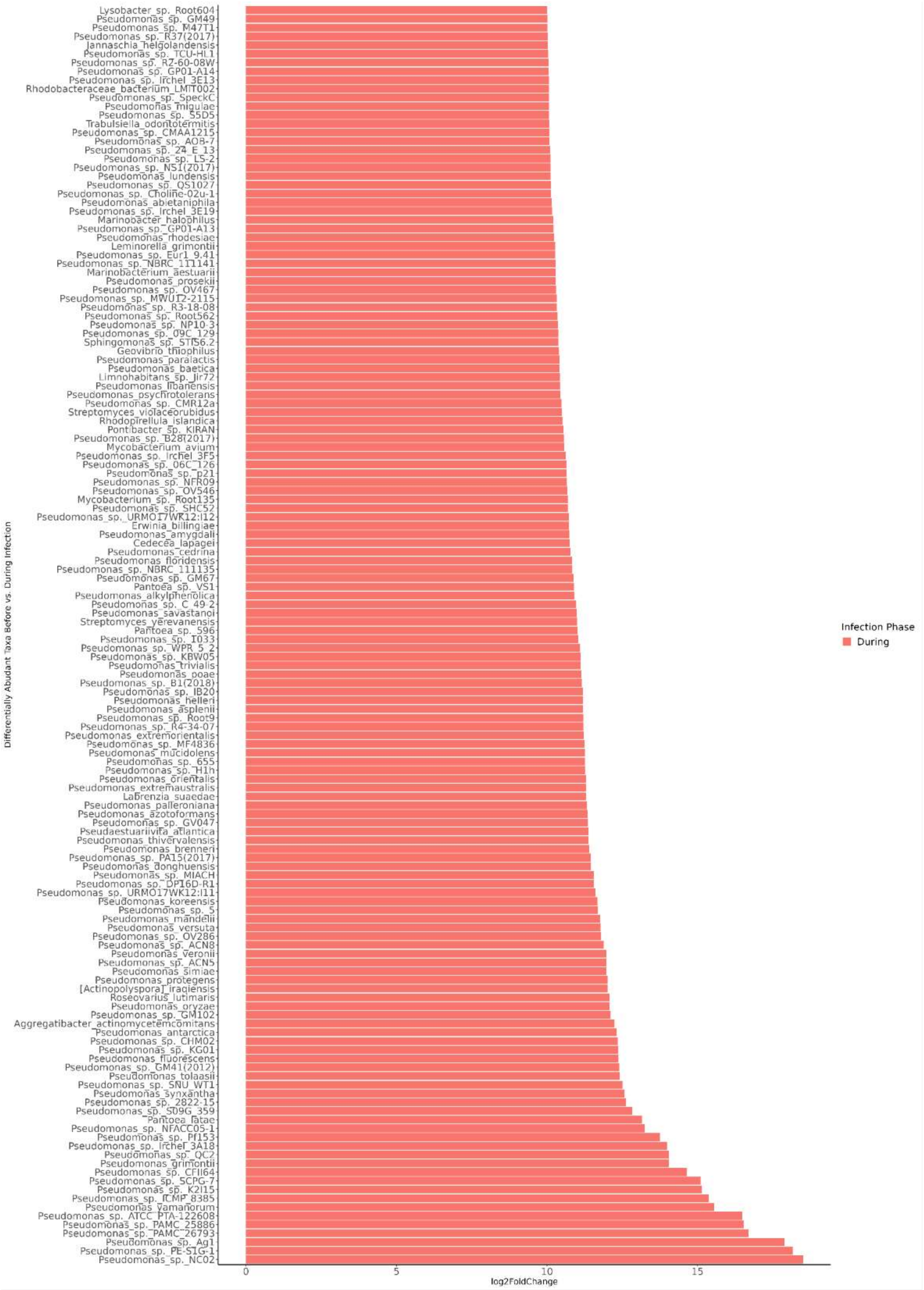
Differentially Abudant Taxa Before vs. During Infection
Lysobacter_sp._Root604
Pseudomonas_sp._GM49
Pseudomonas_sp._M47T1
Pseudomonas_sp._R37(2017)
Jannaschia_helgolandensis
Pseudomonas_sp._TCU-HL1
Pseudomonas_sp._RZ-60-08W
Pseudomonas_sp._GP01-A14
Pseudomonas_sp._Irchel_3E13
Rhodobacteraceae_bacterium_LMIT002
Pseudomonas_sp._SpeckC
Pseudomonas_migulae
Pseudomonas_sp._55D5
Trabulsiella_odontotermitis
Pseudomonas_sp._CMAA1215
Pseudomonas_sp._AO8-7
Pseudomonas_sp._24_E_13
Pseudomonas_sp._LS-2
Pseudomonas_sp._NS1(2017)
Pseudomonas_lundensis
Pseudomonas_sp._QS1027
Pseudomonas_sp._Choline-02u-1
Pseudomonas_abietaniphila
Pseudomonas_sp._Irchel_3E19
Marinobacter_halophilus
Pseudomonas_sp._GP01-A13
Pseudomonas_rhodesiae
Leminorella_grimontii
Pseudomonas_sp._Eur1_9.41
Pseudomonas_sp._NBRC_111141
Marinobacterium_aestuarii
Pseudomonas_prosekii
Pseudomonas_sp._OV467
Pseudomonas_sp._MWU12-2115
Pseudomonas_sp._R3-18-08
Pseudomonas_sp._Root562
Pseudomonas_sp._NP10-3
Pseudomonas_sp._09C_129
Sphingomonas_sp._STIS6.2
Geovibrio_thiophilus
Pseudomonas_paralactis
Pseudomonas_baetica
Limnohabitans_sp._Jir72
Pseudomonas_libanensis
Pseudomonas_psychrotolerans
Pseudomonas_sp._CMR12a
Streptomyces_violaceorubidus
Rhodopirellula_islandica
Pontibacter_sp._KIRAN
Pseudomonas_sp._B28(2017)
Mycobacterium_avium
Pseudomonas_sp._Irchel_3F5
Pseudomonas_sp._06C_126
Pseudomonas_sp._p21
Pseudomonas_sp._NFR09
Pseudomonas_sp._OV546
Mycobacterium_sp._Root135
Pseudomonas_sp._SHC52
Pseudomonas_sp._URMO17WK12:I12
Erwinia_billingiae
Pseudomonas_amygdali
Cedecea_lapagei
Pseudomonas_cedrina
Pseudomonas_floridensis
Pseudomonas_sp._NBRC_111135
Pseudomonas_sp._GM67
Pantoea_sp._VS1
Pseudomonas_alkylphenolica
Pseudomonas_sp._C_49-2
Pseudomonas_savastanoi
Streptomyces_yerevanensis
Pantoea_sp._596
Pseudomonas_sp._I033
Pseudomonas_sp._WPR_5_2
Pseudomonas_sp._KBW05
Pseudomonas_trivialis
Pseudomonas_poae
Pseudomonas_sp._B1(2018)
Pseudomonas_sp._IB20
Pseudomonas_helleri
Pseudomonas_asplenii
Pseudomonas_sp._Root9
Pseudomonas_sp._R4-34-07
Pseudomonas_extremorientalis
Pseudomonas_sp._MF4836
Pseudomonas_mucidolens
Pseudomonas_sp._655
Pseudomonas_sp._H1h
Pseudomonas_orientalis
Pseudomonas_extremaustralis
Labrenzia_suaedae
Pseudomonas_palleroniana
Pseudomonas_azotoformans
Pseudomonas_sp._GV047
Pseudaestuariivita_atlantica
Pseudomonas_thivervalensis
Pseudomonas_brenneri
Pseudomonas_sp._PA15(2017)
Pseudomonas_donghuensis
Pseudomonas_sp._MIACH
Pseudomonas_sp._DP16D-R1
Pseudomonas_sp._URMO17WK12:I11
Pseudomonas_koreensis
Pseudomonas_sp._5
Pseudomonas_mandelii
Pseudomonas_versuta
Pseudomonas_sp._OV286
Pseudomonas_sp._ACN8
Pseudomonas_veronii
Pseudomonas_sp._ACN5
Pseudomonas_simiae
Pseudomonas_protegens
[Actinopolyspora]_iraqiensis
Roseovarius_lutimaris
Pseudomonas_oryzae
Pseudomonas_sp._GM102
Aggregatibacter_actinomycetemcomitans
Pseudomonas_antarctica
Pseudomonas_sp._CHM02
Pseudomonas_sp._KG01
Pseudomonas_fluorescens
Pseudomonas_sp._GM41(2012)
Pseudomonas_tolaasii
Pseudomonas_sp._SNU_WT1
Pseudomonas_synxantha
Pseudomonas_sp._2822-15
Pseudomonas_sp._S09G_359
Pantoea_latae
Pseudomonas_sp._NFACC05-1
Pseudomonas_sp._Pf153
Pseudomonas_sp._Irchel_3A18
Pseudomonas_sp._QC2
Pseudomonas_grimontii
Pseudomonas_sp._CFII64
Pseudomonas_sp._SCPG-7
Pseudomonas_sp._K2I15
Pseudomonas_sp._ICMP_8385
Pseudomonas_yamanorum
Pseudomonas_sp._ATCC_PTA-122608
Pseudomonas_sp._PAMC_25886
Pseudomonas_sp._PAMC_26793
Pseudomonas_sp._Ag1
Pseudomonas_sp._PE-S1G-1
Pseudomonas_sp._NC02
0
5
10
15
log2FoldChange
Infection Phase
During

Figure S4. Number of differentially abundant species found when comparing gut microbiomes before versus during norovirus AGE. Taxa demonstrating a >10 log2 fold change and adjusted p-values < 0.01 are shown in the figure. In all, 1,172 differentially abundant species were found at the p-adj < 0.05 level. Participant A was excluded from the species-level differential abundance analysis since the gut microbiome was not disrupted during norovirus AGE.
